# 

**Published:** 1903-04

**Authors:** 


### BOOKS RECEIVED.

Diseases of the Stomach. A Textbook for Practitioners and Students. By Max Einhorn, M. D., Professor in Clinical Medicine at the New York Post-Graduate Medical School and Hospital; Visiting Physician to the German Dispensary. Octavo, pp. 551. Third revised edition. Illustrated. New York: William Wood & Co. 1903. [Price, $3 50.]

Report of the Commissioner of Education for the year 1900-1901. Volume II. Washington: Government Printing Office. 1902.

The Internal Secretions and the Principles of Medicine. By Charles E. de M. Sajous, M. D., Fellow of the College of Physicians of Philadelphia; formerly Lecturer on Laryngology in Jefferson Medical College, and Professor of Laryngology and Dean of the Faculty in the Medico-Chirurgical College. Volume First. Octavo, pp. 826. With 42 illustrations. Philadelphia: F. A. Davis Co. 1903.

Proceedings of the American Medico-Psychological Association at the Fifty-eighth Annual Meeting, held at Montreal, P. Q., June 17-20, 1902. C. B. Burr, M. D., Secretary. Published by American Medico-Psychological Association. 1902.

The American Year Book of Medicine and Surgery for 1903. A yearly Digest of Scientific Progress and Authoritative Opinions in all branches of Medicine and Surgery, drawn from journals, monographs and textbooks of the leading American and foreign authors and investigators. Arranged, with critical editorial comments, by eminent American specialists, under the editorial charge of George M. Gould, A. M., M. D. In two volumes—Volume I., including General Medicine. Octavo, 700 pages, fully illustrated. Volume II., General Surgery. Octavo, 670 pages, fully illustrated. Philadelphia, New York and London: W. B. Saunders & Co. 1903. [Per volume, cloth, $3.00 net; half morocco, $3.75 net.]

Harrington's Hygiene. A Manual of Practical Hygiene for Students, Physicians and Health Officers. By Charles Harrington, M. D., Assistant Professor of Hygiene in the Medical School of Harvard University. New (2d) edition, revised and enlarged. In one octavo volume of 755 pages, illustrated with 113 engravings and 12 full-page plates in colors and monochrome. Philadelphia and New York: Lea Brothers & Co. 1903. [Cloth, $4.25 net.]

Progressive Medicine. Fifth annual series. Volume I., March, 1903. A Quarterly Digest of Advances, Discoveries and Improvements in the Medical and Surgical Sciences. Edited by Hobart Amory Hare, M. D., Professor of Therapeutics and Materia Medica in the Jefferson Medical College of Philadelphia.

Octavo, 450 pages, illustrated. Philadelphia and New York: Lea Brothers & Co. [Per volume, $2.50, by express prepaid; per annum, in four cloth-bound volumes, $10.00.]

Clinical Treatises on the Pathology and Therapy of Disorders of Metabolism and Nutrition. By Prof Dr. Carl von Noorden, Physician-in-Chief to the City Hospital, Frankfurt a. M. Authorised American edition, translated under the direction of Boardman Reed, M. D., Professor of Diseases of the Gastrointestinal Tract, Hygiene and Climatology, Department of Medicine, Temple College; Physician to the Samaritan Hospital, Philadelphia. Part III. Membranous Catarrh of the Intestines. By Prof. Dr. Carl von Noorden. With the collaboration of Dr. Carl Dapper. Duodecimo, pp. 64. New York: E. B. Treat & Co. 1903. [Price, 50 cents.]

The Surgical Diseases of the Genitourinary Organs. By E. L. Keyes, A. M., M. D., LL.D., Consulting Surgeon to the Bellevue, and the Skin and Cancer Hospitals; Surgeon to St. Elizabeth Hospital, New York. And E. L. Keyes, Jr., A. B., M. D., Ph. D., Lecturer on Genitourinary Surgery, New York Polyclinic Medical School and Hospital; Surgeon to the Out-Patient Department, St. Vincent's Hospital. A revision of Van Buren and Keyes's Textbook. Octavo, pp. 843. With 174 illustrations in the text and 10 plates, 8 of which are colored. New York and London: D. Appleton & Co. 1903. [Price, $5.00.]

Surgical Anatomy. A Treatise on Human Anatomy inits Application to the Practice of Medicine and Surgery. By John B Deaver, M. D., Surgeon-in-Chief to the German Hospital, Philadelphia. In three volumes. Illustrated by 499 plates, nearly all drawn for this work from original dissections. Volume III. Abdomen; pelvic cavity; lymphatics of the abdomen and pelvis; thorax; lower extremity. Philadelphia: P. Blakiston's Son & Co. 1903. [Price: cloth, $21.00; full sheep, $24.00; half Russia, gilt, marbled edges, $27.00 net.]

